# Transesterification of Sunflower Oil over Waste Chicken Eggshell-Based Catalyst in a Microreactor: An Optimization Study

**DOI:** 10.3390/mi12020120

**Published:** 2021-01-23

**Authors:** Stefan Pavlović, Gordana Šelo, Dalibor Marinković, Mirela Planinić, Marina Tišma, Miroslav Stanković

**Affiliations:** 1 Institute of Chemistry, Technology and Metallurgy, National Institute for the Republic of Serbia, University of Belgrade, Njegoševa 12, 11 000 Belgrade, Serbia; stefan.pavlovic@ihtm.bg.ac.rs (S.P.); dalibor@ihtm.bg.ac.rs (D.M.); mikastan@nanosys.ihtm.bg.ac.rs (M.S.); 2 Faculty of Food Technology Osijek, Josip Juraj Strossmayer University of Osijek, Osijek, F. Kuhača 18, 31 000 Osijek, Croatia; gordana.selo@ptfos.hr (G.Š.); mirela.planinic@ptfos.hr (M.P.)

**Keywords:** microreactor, chicken eggshell-based catalyst, transesterification, biodiesel, statistical process optimization, Box–Behnken design

## Abstract

The statistical experimental design (DoE) and optimization (Response Surface Methodology combined with Box–Behnken design) of sunflower oil transesterification catalyzed by waste chicken eggshell-based catalyst were conducted in a custom-made microreactor at 60 °C. The catalyst was synthesized by the hydration–dehydration method and subsequent calcination at 600 °C. Comprehensive characterization of the obtained catalyst was conducted using: X-ray powder diffractometry (XRD), X-ray fluorescence (XRF), Fourier-transform infrared (FT-IR) spectroscopy, scanning electron microscopy (SEM), N_2_ physisorption, and Hg-porosimetry. Structural, morphological, and textural results showed that the obtained catalyst exhibited high porosity and regular dispersity of plate-like CaO as an active species. The obtained optimal residence time, catalyst concentration, and methanol/oil volume ratio for the continuous reaction in microreactor were 10 min, 0.1 g g^−1^, and 3:1, respectively. The analysis of variance (ANOVA) showed that the obtained reduced quadratic model was adequate for experimental results fitting. The reaction in the microreactor was significantly intensified compared to a conventional batch reactor, as seen through the fatty acid methyl esters (FAMEs) content after 10 min, which was 51.2% and 18.6%, respectively.

## 1. Introduction

The worldwide environmental problems (global warming, climate change, biodiversity concern, and different types of pollution) caused by the use of fossil fuels and solid waste disposal lead to intensive research in the field of sustainable processes of chemical and fuel production [1,2]. Biodiesel is one of these alternative fuels. It is a liquid, biodegradable and non-toxic fuel due to the extremely poor sulphur content. It can be used either in pure form or in a mixture with diesel fuel in diesel engines, leading to a remarkable reduction in the amount of unburned hydrocarbons, emissions of carbon dioxide, carbon monoxide, and suspended particles from exhaust gases [3].

Today’s industrial biodiesel production is mainly based on chemically catalyzed transesterification with homogeneous basic or acidic catalysts using edible vegetable oils as feedstocks. Beside several issues, such as the “food versus fuel” feedstock dilemma and the use toxic and environmentally unfriendly catalysts causing soap generation, wastewater accumulation and equipment corrosion (acid catalyst), another problem is related to the low efficiency of the commonly used modern bioreactor systems [4]. Based on the zero waste concept, many studies are focused on the investigation of different waste-based catalytic systems [5,6,7] and waste feedstocks [8,9] for sustainable biodiesel production. A special contribution is pointed out in the development of continuous reactor systems for heterogeneously catalyzed reactions, either by acidic, basic or enzymatic catalysts [10]. Major drawbacks of commonly commercially used batch reactors for biodiesel production are long reaction time, low energy efficiency, problematic selectivity, slow response to change in process parameters, heat and mass transfer issues (especially with heterogeneous catalyst), etc. Some novel reactors, such as microchannel reactors, have been developed to achieve high mixing performance and improve mass transfer and, therefore, increase reaction yield [11]. Microreactors present microfluidic systems in which chemical reactions are performed in a controlled manner within a network of microchannels (widths and depths range from tens to hundreds of micrometers), where the fluid flow is laminar and mass and heat transfer are strongly enhanced [12,13]. These reactors have been shown to be suitable for optimization of many heterogeneously-catalyzed synthetic procedures because of their positive effect on transfer phenomena in the overall reaction, like glucose oxidation, Knoevenagel condensation, alkene epoxidation, enzymatic hydrolysis, esterification, etc. [14]. The main feature of microreactors is their high surface-to-volume ratio in the range of 10,000–50,000 m^2^ m^−3^ compared to conventional laboratory and production reactors where this ratio is usually 100 m^2^ m^−3^ and seldom exceeds 1000 m^2^ m^−3^ [14]. The small diameters of the reactor channels enable a short radial diffusion time, leading to a narrow residence time distribution. This could be advantageous in transesterification reactions due to the possibility of achieving high selectivity to the desired product, i.e., biodiesel. This technology can achieve rapid and high reaction rates due to the high surface-to-volume ratio and short diffusion distance, thus intensifying the transesterification process [15]. Another additional important aspect is the easier scale-up by multiplying the number of microreactor units without change of the channel geometry [16,17]. In the process of biodiesel production, time, temperature, type and amount of used catalyst, mixing intensity, alcohol and oil type and their molar ratio are the effective parameters of reaction efficiency. Using microreactors, due to the high surface-to-volume ratio, the oil and alcohol feeds are mixed in a micro-sized channel, resulting in better mass transfer between two immiscible phases [18].

In recent years, researchers’ attention has been drawn to the development of heterogeneously catalyzed biodiesel production in microreactors [19]. Many disadvantages of the conventional homogeneous base catalyzed process, which are reflected in high catalyst and operational costs, large wastewater processing, product purification, and saponification of free fatty acids (FFAs), could be overcome by using solid catalysts, especially non-supported or supported metal oxides of alkali-earth metals, such as calcium, strontium, and magnesium. Among these catalysts, calcium oxide has attracted attention as a catalyst for the production of biodiesel due to the abundance of its natural or waste sources such as shells (eggs, oysters, and clams) [20,21], bones [22,23] and lime [24]. Such catalysts exhibit suitable catalytic activity and stability under moderate reaction conditions. In addition, their low cost, non-corrosive effect, and simple separation from the reaction mixture present important advantages of this type of catalyst. Mohadesi et al. [2] studied biodiesel production in a semi-industrial pilot of microreactors using KOH/Clinoptilolite catalyst, whereby the highest biodiesel purity (97.5%) was obtained at moderate reaction conditions (temperature of 65 °C, catalyst concentration of 8.1%, and methanol/oil volume ratio of 2.25:1) for the residence time of 13.4 min. On the other hand, biodiesel production in a microreactor with kettle limescale deposit as a catalyst was efficient, whereby the highest biodiesel purity of 97.16% was achieved at 60 °C and a methanol/oil volume ratio of 2.15:1. In order to improve miscibility in this heterogeneous system, acetone at a concentration of 13.95% was used as a co-solvent [25]. Aghel et al. [26] pointed out the high potential of natural material based on calcium, such as water plant sedimentation in transesterification of soybean oil in a microreactor. It is shown that the highest biodiesel purity of 93.14% was achieved at optimal reaction conditions (temperature of 60 °C, catalyst concentration of 0.0837 g g^−1^, methanol/oil volume ratio of 1:1.89, and residence time of 10 min).

The main objectives of the present study were to synthesize and characterize an efficient waste chicken eggshell-based catalyst for the sunflower oil transesterification, and to optimize the key reaction parameters (catalyst concentration, methanol/oil volume ratio, and residence time) in the custom-made microchannel reactor in a time-saving manner using statistical experimental design (DoE). The Box–Behnken design combined with response surface methodology will provide a mathematical model in the form of a quadratic equation which, after ANOVA assessment, will provide insight, not only into the optimal conditions for performing the reaction but also into the influence of individual process parameters and their interactions on the reaction yield.

## 2. Materials and Methods

### 2.1. Materials

#### Chemicals

Raw chicken eggshells (ESs) were gathered in the household. Edible sunflower oil (Zvijezda, Croatia) was purchased from a local supermarket. Methanol (99.99%, GC quality, Acros Organics) was used as a reagent. 2-propanol and n-hexane were HPLC grade and used as solvents in high-performance liquid chromatography (HPLC) analysis of FAMEs.

### 2.2. Methods

#### 2.2.1. Catalyst Preparation

Raw chicken eggshells (ES-R) were washed, dried, grounded, and sieved. Such prepared ESs were calcined at 900 °C during 4 h (heating rate of 5 °C min^−1^) and the obtained sample was denoted as ES-900. In order to improve textural and basicity properties, the calcined ESs were underwent the hydration–dehydration procedure described elsewhere [1]. The hydration was performed in the three-neck round bottom flask at 60 °C, 6 h with the solid/liquid ratio of 1:5. After hydration, the suspension was filtered and dried at 110 °C overnight. The obtained powder was calcined at 600 °C for 4 h (heating rate of 5 °C min^−1^) and such prepared sample was denoted as ES-HC-600, which was used as a final catalyst. The ES-HC-600 was kept sealed in vials and stored in a desiccator until catalytic tests.

##### Catalyst Characterization

The chemical composition of the raw and synthesized samples was analyzed using XRF analysis equipment (EDX-8000 energy dispersive X-ray fluorescence spectrometer, Shimadzu, Kyoto, Japan). The identification of a crystalline structure was carried byXRD, (D8 Endeavor diffractometer, Bruker, Billerica, MA, USA) over the angular range of 10–90° (2*θ*) at a scanning rate 1° min^−1^ with a step size of 0.02°, using CoKα radiation (*λ* = 0.178896 nm). FT-IR spectra were recorded using a Shimadzu IRAffinity-1 Fourier-transform infrared spectrophotometer (Attenuated Total Reflection-MIRacle 10) in the wavenumber range of 4000–400 cm^−1^ using 64 scans at 8 cm^−1^ resolution. The surface morphology was analyzed by a field emission SEM (Tescan MIRA3 XMU, Tescan, Brno, Czech Republic). Prior to imaging, the dried powder samples were sputter-coated with a thick, uniform layer of Au/Pd alloy. The specific surface area was calculated from the nitrogen adsorption/desorption isotherms obtained at 77 K in an accelerated surface area and porosimetry (ASAP) instrument (ASAP 2020, Micromeritics, Norcross, GA, USA) using the Brunauer-Emmett-Teller (BET) equation. Hg-porosimetry measurements were performed in the fully automated conventional apparatus Carlo Erba 2000 porosimeter (pressure range: 0.1–200 MPa; pore size diameter range: 7.5–15,000 nm) and data acquisition was carried out using the associated software package Milestone 200 (Carlo Erba, Milano, Italy). The particle size distribution of powder samples was estimated by Hg-porosimetry measurements applying the Mayer-Stowe model.

#### 2.2.2. Transesterification in the Microreactor

**Figure 1:**
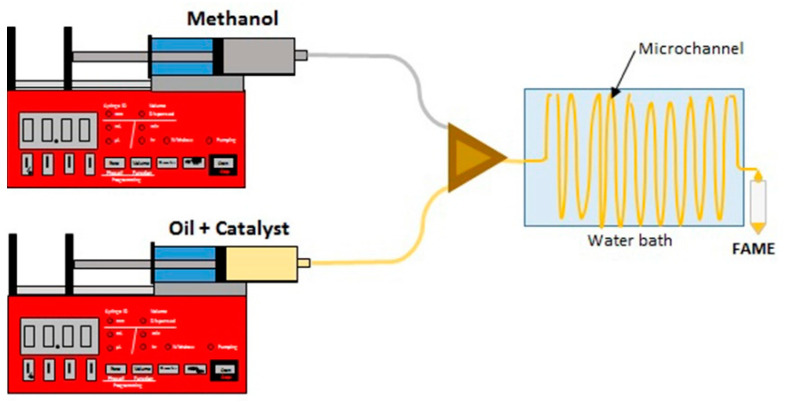
Transesterification microreactor setup.

The transesterification reaction was performed in a custom-made microreactor which was equipped with precise syringe pumps and a thermostated water bath. Figure 1 shows the schematic diagram of the microreactor system.

The microreactor setup had two parts including a T-shaped three-way junction and a Teflon microchannel with an internal diameter of 0.8 mm and length of 800 mm. To prepare oil solutions, it is necessary to calculate specific weights of CaO catalyst and then mix it with oil. The experimental domain is selected as follows: residence time in the range of 4 to 10 min, catalyst concentration in the range of 0.05 to 0.1 g g^−1^, and methanol/oil volume ratio in the range of 1.5:1 to 3:1. The mixture of oil and catalyst was treated by ultrasound for 3 min and then added into the syringe. Two syringe pumps (Son Pump 350) were employed to inject the methanol and mixture of sunflower oil and catalyst separately into the microreactor at different flow rates. The methanol and mixture of sunflower oil and catalyst were combined at a T-shaped junction and then the mixture passed through the Teflon microchannel. According to residence time and methanol to oil volume ratio values, the flow rate was set in the range of 16.96 to 53.01 μL min^−1^ for the mixture of sunflower oil and catalyst and 7.07 to 28.27 μL min^−1^ for methanol, respectively. The reaction temperature was maintained at 60 °C using a water bath (the microreactor was immersed in it). The product was gathered in the beaker placed in an ice-water flask to stop the transesterification reaction and then centrifuged at 10,000 rpm for 10 min (Hermle Z 326K) to separate the FAME’s phase from the rest of the reaction mixture. In order to compare the continuous process with the conventional process in the batch reactor, transesterification of sunflower oil was carried out in a 500 mL three necked spherical glass reactor coupled to a reflux condenser and equipped with a magnetic stirrer. The reactor was immersed in a constant temperature water bath placed on a hotplate (IKA C-MAG HS 7, IKA, Staufen, Germany) coupled with a temperature controller. The reaction mixture was magnetically stirred (850 rpm). Catalytic tests were performed under commonly operational conditions (temperature of 60 °C, methanol/oil molar ratio of 12:1, and catalyst concentration of 8 wt%). The desired amounts of methanol and catalyst were loaded to the reactor and thermostated to the required temperature while agitated (30 min). Separately, sunflower oil was thermostated at the same temperature. After thermostating, the stirrer was turned off and the oil was added to the reactor. Thereafter, the stirrer was switched on at a stirring rate of 850 rpm and the reaction was timed. Samples of the reaction mixture were taken from the reactor during the reaction and centrifuged at 10,000 rpm for 10 min (Hermle Z 326K, HERMLE Labortechnik, Wehingen, Germany) to separate the (FAME) phase from the rest of the reaction mixture.

#### 2.2.3. Design of Experiments and Optimization

Determination of the best reaction conditions can be done by both statistical and experimental optimization [27]. The statistical approach is time-saving because it reduces the number of required experiments to obtain optimal reaction conditions. Moreover, this method allows simultaneous study of the effects of several reaction parameters and their interaction on biodiesel synthesis. Whether batch [24,28,29] or continuous [30,31] processes were investigated, statistical optimization combining response surface methodology with experimental design has been successfully implemented [32]. This methodology, in the present study, is conducted in four steps, as follows. First, the most significant influential factors are selected and their experimental domains of interest are defined based on previous experience. Second, adequate statistical experimental design is selected, based on the constraints of the selected ranges of the influential process factors. So far, for the transesterification reaction catalyzed over CaO-based catalysts’ central composite, full factorial and Box–Behnken designs have been successfully applied [27]. However, in comparative study, the Box–Behnken design emerged as economically advantageous, so that design was adopted in the present study [32]. A polynomial equation with quadratic terms is necessary to determine a function minimum or maximum. The present study defined the following equation as a relation of the FAME content (*y*) with the residence time (*x*_1_), catalyst concentration (*x*_2_), and methanol/oil volume ratio (*x*_3_) as process factors:

(1) $$y=\alpha_{0}+\overset{k}{\underset{i=1}{\sum }}\alpha_{i}x_{i}+\overset{k}{\underset{i=1}{\sum }}\alpha_{ii}x_{i}^{2}+\overset{k}{\underset{1\leq i\leq j}{\sum }}\alpha_{ij}x_{i}x_{j}+\varepsilon$$

where: *α* is the regression coefficient, *k* is the number of variables, and *ε* is the observed residual. The regression coefficients are determined by the multiple nonlinear regression using Design-Expert 11 (Stat-Ease Inc, Minneapolis, MN, USA) software. The third step involved ANOVA analysis of the proposed model quality. The impact of the individual process factors, their mutual interactions and second-order terms are also comprehensively assessed by ANOVA. This analysis is based on the comparison of two variations, the first due to the interchange in the combination of the levels of the factors, and the second due to the unarranged errors inherent to the results of the obtained dependent variable, i.e., FAME content [33]. The ANOVA method is primarily used to evaluate the significance of the developed model. The Fisher distribution test (*F*-test) with a confidence level of 95% is used to assess the significance of the influential factors and their mutual interaction. The fourth and final step implied definition of the optimization criteria and priorities following the objectives of the present experimental study. These criteria are used in determining the optimum levels of the process factors using the developed model equation (Equation (1)). In that manner, the highest FAME yield is ensured in the applied experimental domains. Often, the developed model equation is simplified by excluding the statistically insignificant process terms, thus increasing the accuracy and quality.

#### 2.2.4. Measurement of FAMEs Concentration

The concentration of formed FAMEs was analyzed by the modified Holčapek HPLC method described elsewhere [34]. Before the HPLC analysis, the samples of FAME were diluted with a mixture of *n*-hexane and 2-propanol (5:4 *v/v*) in a ratio of 1:200 and filtered through a 0.45 μm pore size membrane filter.

## 3. Results and Discussion

### 3.1. XRD and XRF Results

**Figure 2:**
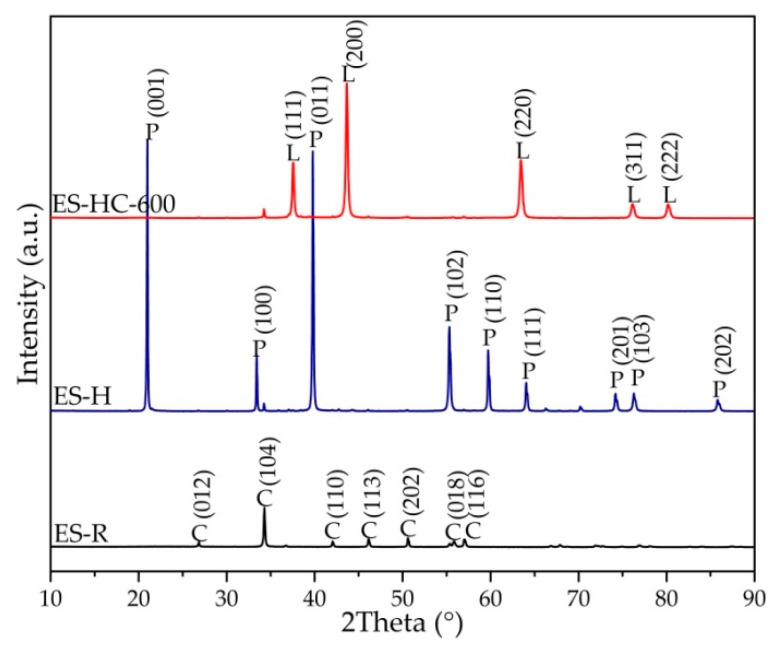
X-ray diffractometry (XRD) patterns of the raw chicken eggshells (ES-R), hydrated ES (ES-H), and catalyst (ES-HC-600); where: C—Calcite; P—Portlandite; and L—Lime.

Figure 2 depicted the crystalline phase composition of ES-R, hydrated ES (ES-H), and synthesized catalyst (ES-HC-600). The X-ray diffractograms of ES sample revealed the typical reflections that can be ascribed to calcium carbonate in the form of calcite (PDF#05-0586). During the synthesis process, calcined ES was converted into a hydrated form, portlandite (PDF#44–1481), in the hydration process. In order to obtain catalytic active calcium oxide form, portlandite was calcined, whereby the active form was obtained at a lower temperature than the conversion temperature of pure carbonate phase. The obtained results are in accordance with the research of Yoosuk et al. [35], which showed that hydration–dehydration is a simple and flexible method for increasing the activity and improving the properties of calcined natural calcite to make them highly suitable for biodiesel production.

The high content of calcium was confirmed by XRF measurements, whereby it was determined that there was a Ca content of 66.5 wt% and 68.7 wt% in the ES-R and ES-900, respectively. In addition, the presence of silicon (0.4 wt%), aluminum (0.7 wt%), potassium (0.1 wt%), sodium (1.9 wt%), and magnesium (0.7 wt%) are noted.

### 3.2. Infrared Spectroscopy Results

**Figure 3:**
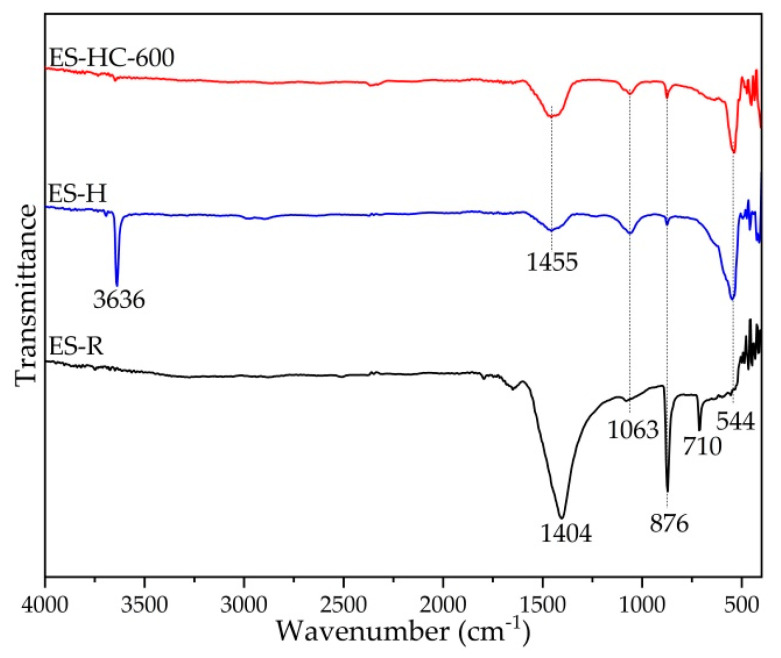
FT-IR spectra of ES-R, ES-H, and ES-HC-600.

Structural information concerning the vibrations of chemical bonds in the molecular units was obtained by FT-IR spectroscopy (Figure 3). The spectrum of ES-R exhibited major absorption bands at 1404 cm^−1^ due to asymmetric stretching of CO_3_^2−^ at 876 cm^−1^ caused by the out-of-plane bend vibration mode of CO_3_^2−^, and at 710 cm^−1^ due to the Ca-O bond and the in-plane bend vibration mode of CO_3_^2−^. The hydrated form of calcined CaO exhibits characteristic wide and low-intensity absorption band at 3636 cm^−1^, which can be ascribed to O-H band vibration. After the heat treatment at 600 °C, portlandite was converted into oxide form, whereby it is evident that the characteristic portlandite absorption band is lost and a new dominant absorption band emerged at 544 cm^−1^, characteristic of Ca-O bond vibration. These results are in accordance with XRD results.

### 3.3. Textural and Morphology Results

**Figure 4:**
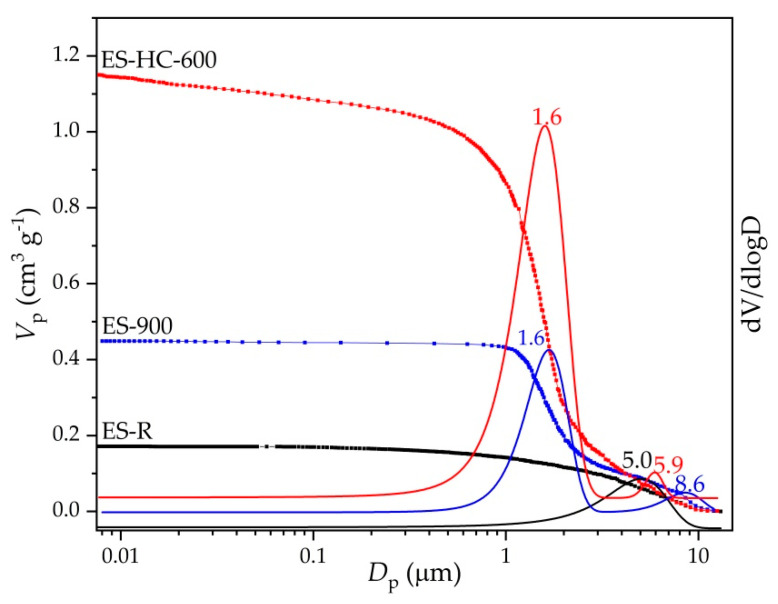
Cumulative volume intrusion and pore size distribution of ES-R, ES-900, and ES-HC-600.

**Figure 5:**
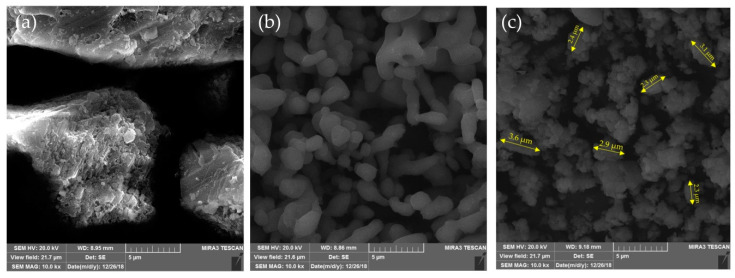
Scanning electron microscope (SEM) micrographs of raw materials and products during catalyst synthesis: (**a**) ES-R, (**b**) ES-900, and (**c**) ES-HC-600.

**Figure 6:**
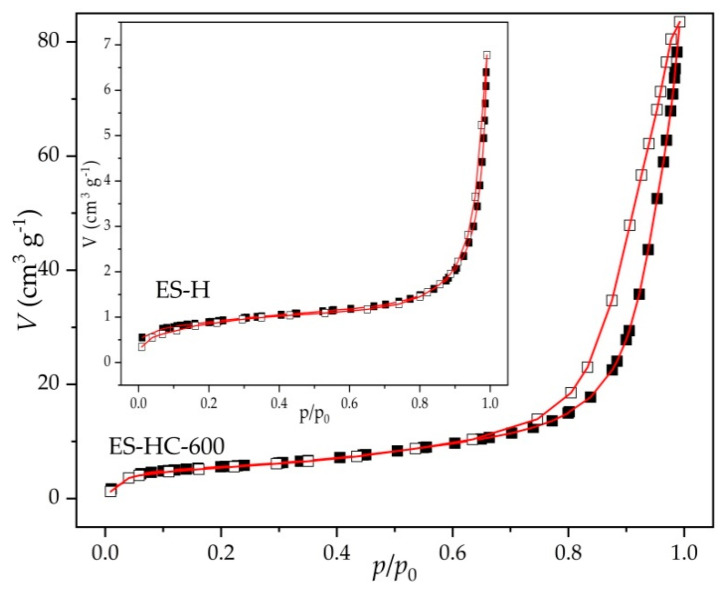
N_2_-adsorption-desorption isotherms of ES-H and ES-HC-600.

The cumulative volume intrusion and pores’ size distribution curves for raw and synthesized samples are shown in Figure 4. ES-R exhibits unimodal distribution with a sharp uptake for pore diameters centered at about 5.0 μm. This result is in accordance with SEM micrographs (Figure 5), which indicated the compact structure of ES-R with low porosity (29.8 vol. %) and the dominant presence of macropore. The thermal treatment led to an increase in porosity and pore volume with a sharp uptake centered at 1.6 μm. Further, hydration–dehydration led to a better definition of pore and channel networks. It can be seen that ES-HC-600 exhibited high porosity (73.6 vol. %) with a well-defined pore structure that is adequate for the present reaction, where reactants are large organic molecules, such as triacylglycerol. The nitrogen adsorption experiments (BET analysis) were in accordance with Hg-porosimetry measurements (Table 1). The ES-R and ES-900, in comparison with the hydrated–dehydrated form of CaO, had a smaller specific surface area, which agreed with the reported data [20]. In accordance with International Union of Pure and Applied Chemistry (IUPAC) classification [36], the hysteresis loop corresponded to type III (Figure 6), characteristic of the non-rigid aggregate of plate-like structures. Such distinctive plate-like surface morphology is seen on the SEM micrographs (Figure 5).

Textural properties of precursors and prepared catalysts are presented in Table 1. The results showed that the thermal and re-hydration treatment leads to significant improvement in the porous structure (specific surface and pore diameter). Developed porosity catalyst was obtained from slightly porous precursors, whereby the specific surface was shifted from less than 1 m^2^ g^−1^ to 19.3 m^2^ g^−1^. It is even more drastic with a specific pore volume; using the Barrett–Joyner–Halenda (BJH) method, the specific pore volume was increased from 0.9 mm^3^ g^−1^ to 121.2 mm^3^ g^−1^ from ES-R to ES-HC-600, respectively.

**Figure 7:**
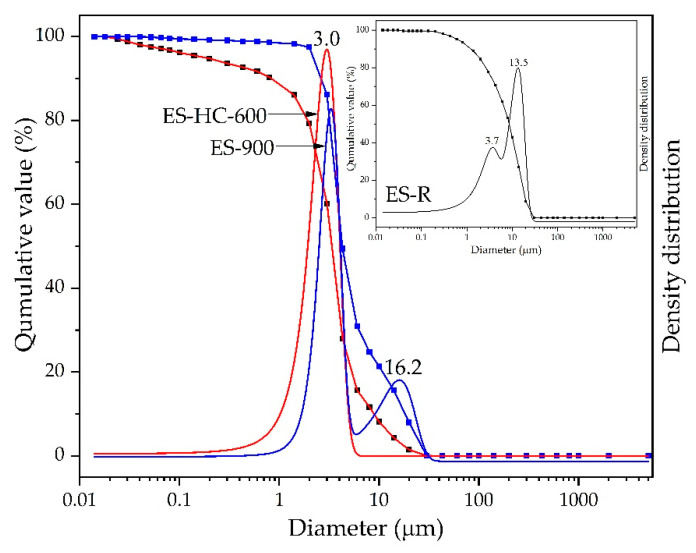
Particle size distribution of ES-R, ES-900, and ES-HC-600.

The particle size density distribution curves for the ES-R, ES-900, and ES-HC-600 samples presented in Figure 7 were uniform. The majority of the particles in all three samples were in the diameter range of 0.02 µm to 30 µm, but the average particle diameter was reduced with the continuation of the synthesis process. ES-R showed an average particle diameter of 12.2 µm; by calcination at 900 °C (ES-900), the average diameter decreased to 11.4 µm, while after the hydration–dehydration process and subsequent calcination at 600 °C (ES-HC-600), the average particle size was 3.0 µm. This average value of particle size in the catalyst ES-HC-600 is clearly visible and confirmed on the SEM micrograph (Figure 5c). It is obvious that hydration–dehydration showed a positive effect on particle formation and, coupled with moderate calcination conditions, particle size remains low. As the catalyst particles are at least two orders of magnitude smaller than the diameter of the microreactor channel, there was no formation of catalyst plugs and clogging of the microchannels, which was visually confirmed.

### 3.4. Statistical DoE

The transesterification of sunflower oil over a modified chicken eggshell-based catalyst in the microreactor was statistically modeled and optimized using the response surface methodology in combination with the three-level Box–Behnken design. The reaction temperature of 60 °C was selected because, at atmospheric pressure, the highest FAME yields were obtained in reactions using CaO-based catalysts at temperatures close to the boiling point of methanol [27]. The process influential factors (residence time, catalyst concentration, and methanol/oil volume ratio) were adopted based on their major impact on the FAME yield in the continuous transesterification reaction [30,31].

#### 3.4.1. Regression Modeling

The results of the applied experimental design involving 15 runs are presented in Table 2. In addition, the actual and predicted FAME content and relative deviation of each run are also shown.

The FAME content was firstly correlated with the adopted influential process factors through the full quadratic equation, with high quality of fit statistics (*R*^2^ = 0.97, *R*_adj_^2^ = 0.93, *R*_pred_^2^ = 0.64, coefficient of variation (*C.V.*) = 23.18%, and Adequate precision = 16.39). The ANOVA analysis of the full regression model showed that only the interaction between residence time and methanol/oil volume ratio (*x*_1_*x*_3_) and catalyst concentration and methanol/oil molar ratio (*x*_2_*x*_3_), as process terms, were statistically non-significant (*p*-value ≥ 0.1). All other terms, whether single, quadratic, or interaction, were statistically significant and, therefore, were introduced in the reduced regression model (Equations (2) and (3)).

The obtained reduced quadratic model equations with codded and actual factors are presented by following regression equations, respectively:

(2) $$y=7.47+10.7\cdot x_{1}+12.59\cdot \;x_{2}+2.82\;\cdot \;x_{3}+7.19\cdot x_{1}x_{2}+4.14\cdot x_{1}^{2}+6.9\cdot \;x_{2}^{2}+3.82\cdot x_{3}^{2}$$

and

(3) $$y=105.59-10.06\cdot x_{1}-1822.45\cdot x_{2}-26.8\cdot x_{3}+95.8\cdot x_{1}x_{2}+0.46\cdot x_{1}^{2}\;+11,037.33\cdot x_{2}^{2}+6.79\cdot x_{3}^{2}$$

**Figure 8:**
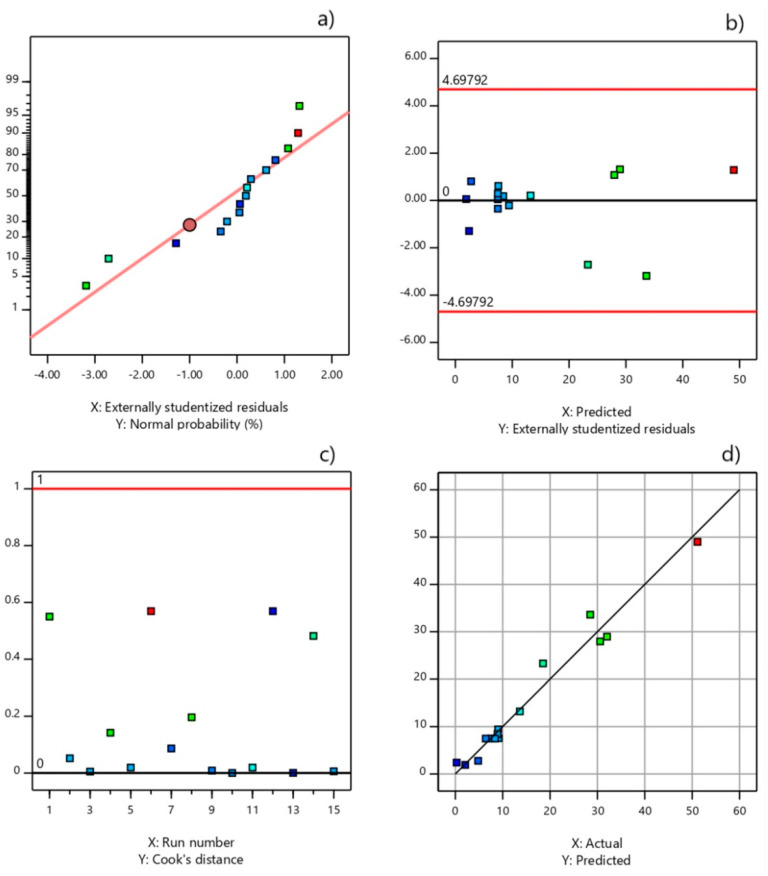
Statistical graphic evaluation of the reduced quadratic model: (**a**) normal probability, (**b**) model outliers, (**c**) Cook’s distance, and (**d**) actual and predicted fatty acid methyl esters (FAME) content plot.

The quality of the fit of the experimental data using the reduced quadratic model was assessed based on several statistical criteria (Table 3). The reduced quadratic model possessed slightly more favorable lack of fit and *C.V.* than the full model, but the *C.V.* value being higher than 10% indicated that the reproducibility of both models can be marked as uncertain in the experimental domain employed. While the full quadratic equation was aliased, its reduced quadratic model was proven to be significant, with an *F*-value of 32.41, a *p*-value < 0.0001, and a non-significant lack of fit (*p* = 0.0558) (Table 3). The coefficient of determination (*R*^2^) value of 0.97 showed the goodness of fit of the derived reduced quadratic model, as it can explain 97% of FAME content variation, whereas only 3% of the variation arises from the uncontrolled factors. The predicted *R*^2^ of 0.84 is in reasonable agreement with the adjusted *R*^2^ of 0.94; i.e., the difference was less than 0.2, thus highlighting a good predictive ability of the proposed reduced quadratic model. The value of the model’s adequate precision was much higher than the critical value of 4 (18.64), showing that the model was adequate for predicting FAME content in the applied experimental domain. The acceptable value of the mean relative percentage deviation (MRPD) of ±11.3% demonstrated the adequate agreement between the actual experimental and predicted data (the relative deviation of experimental run number 12 is not taken into account due to the fact that it deviated too excessively from all other values, which can be explained by its having the lowest value of FAME content, of only 0.28%, of all the experiments). The adequacy of the proposed reduced model for predicting FAME content was assessed using the diagnostic statistical graphs presented in Figure 8. The normal probability plot revealed that the residuals satisfactorily followed the normal distribution, thus confirming that the proposed model satisfied the ANOVA assumptions. The Cook’s distance values were lower than 0.56 (limit value was 1.0), indicating that there was no outlier in the analyzed results. This conclusion is confirmed also by the externally studentized residuals, which are all located between the limit values of ±4.698. Finally, it was found that the use of a reduced quadratic equation was justified, taking into account all statistical indicators of regression quality.

The catalyst concentration (*x*_2_) had the highest *F*-value, which means that it affected the FAME content more significantly than the other two process factors. The residence time (*x*_1_) also had a significant impact, while methanol/oil volume ratio (*x*_3_) had a marginal impact. Such behavior, i.e., the small to negligible influence of the methanol/oil molar ratio, has been noticed for the methanolysis of waste lard in continuous reactor catalyzed by KOH [30]. Although the present study does not demonstrate this, the influence of the alcohol/oil molar ratio on the FAME yield appears to be more complex and dependent on other factors, so that it can also positively affect the FAME content [37]. The positive effects of catalyst concentration on FAME content in the present study are expected to be due to an increase in the number of active catalytic sites, but care should be taken because an excessive increase in catalyst content has been observed to increase the density of the reaction mixture below the critical point and thus make the mass transfer more difficult [27]. The increase in residence time resulted in higher FAME content due to a longer contact time between the two immiscible reactants and the catalyst surface, but in this case, the economics of the process should be taken into account. The Equation (3) had one significant two-factor interaction term affecting FAME content, which involves residence time and catalyst concentration (*x*_1_*x*_2_). The significance of this term in the model was higher than the quadratic terms. Among them, the quadratic term of catalyst concentration (*x*_2_^2^) had higher influence, following the quadratic terms of residence time (*x*_1_^2^), and finally the quadratic term of methanol/oil volume ratio (*x*_3_^2^), with the lowest influence. This arrangement is not surprising knowing the influences of the individual process factors.

#### 3.4.2. Model Validation and Optimization of FAME Conversion

The highest FAME content can be achieved under the optimal reaction conditions. To determine the optimal process parameters’ values requires the setting of certain optimization criteria and priorities. The optimization aim was to maximize FAME content while the process parameters (residence time, catalyst concentration, and methanol/oil volume ratio) remained minimized within the applied experimental domain. In terms of optimization priorities, the highest was given to the FAME content, the middle priority was attached to the catalyst concentration and residence time, while the lowest priority was given to the methanol/oil volume ratio.

**Figure 9:**
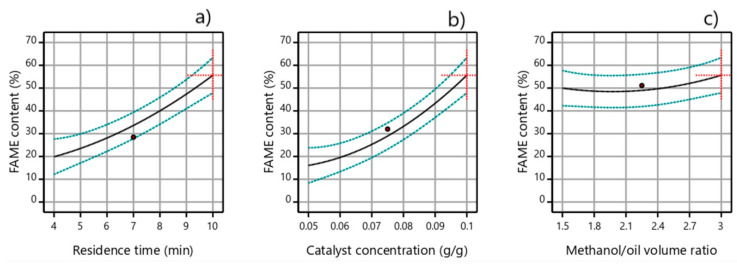
Perturbation plots of FAME content versus (**a**) residence time, (**b**) catalyst concentration, and (**c**) methanol/oil volume ratio (60 °C, catalyst concentration of 0.1 g g^−1^, residence time of 10 min, and methanol/oil volume ratio of 3:1).

Perturbation plots of FAME content concerning the changing of one factor over its range while holding all the other factors constant are presented in Figure 9. It was evident that the residence time and catalyst concentration had a higher influence than the methanol/oil volume ratio. However, when these two process factors are compared in the applied experimental domain, the influence of the catalyst concentration on FAME content (Figure 9b) was slightly higher than that of the residence time (Figure 9a). The change of the catalyst concentration from 0.05 g g^−1^ to 0.1 g g^−1^ increased the FAME content from 16.1% to 55.6%, while the change of the residence time from 4 min to 10 min increased the FAME content from 20.0% to 55.6%. The plot in Figure 9c confirmed that the methanol/oil volume ratio in the range from 1.5 to 3 had little influence on FAME content.

The influence of molar ratio on FAME yield observed in the literature in heterogeneously catalyzed (CaO-based catalyst) batch systems is contradictory. The molar ratio influence went from very low [38], through moderate [39], to significant [40]. The continuous heterogeneously catalyzed reactor systems appear to have more regular behaviors. In the tubular reactor packed with the CaO catalyst, Miladinović et al. [31] reported a low molar ratio and residence time influence. Mohadesi et al. [2], using KOH/Clinoptilolite catalyst in a microreactor, observed also the very low influence of molar ratio, unlike the influence of residence time, which was highest, while the influence of catalyst concentration was moderate to low. Aghel et al. [26] came to a congruent observation using a CaO-based catalyst in a microreactor. On the other hand, Shrimal et al. [41] reported the high influence of methanol/oil molar ratio on FAME yield in a narrow experimental range (from 1:7 to 1:9). It should be noted that the reaction was homogeneously catalyzed in a microreactor system. The moderate or high influence of residence time and catalyst concentration on FAME yield observed in the present study was consistent with the previous studies conducted on comparable systems [2,26,30,32].

**Figure 10:**
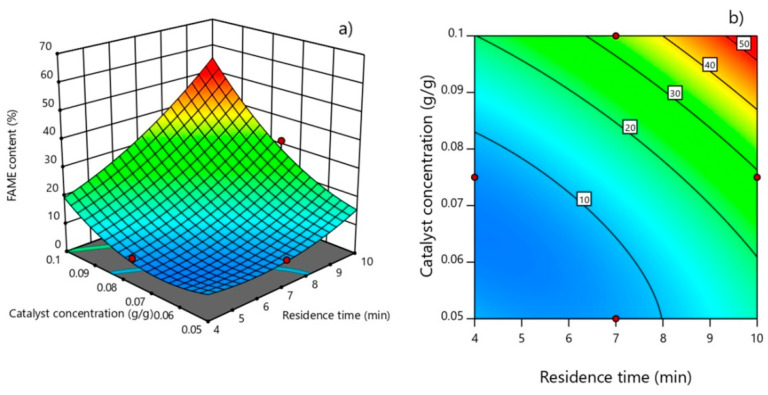
Response (**a**) surface and (**b**) contour plot of FAME content as a function of catalyst concentration and residence time (60 °C, catalyst concentration of 0.1 g g^−1^, the residence time of 10 min, and methanol/oil volume ratio of 3:1).

The interaction contour and 3D plots (residence time versus catalyst concentration) that resulted from the developed reduced quadratic model depicted the increase in the FAME content with an increase in both residence time and catalyst concentration, so that a maximum FAME content of 55.6% is reached at the maximum values of these two process factors in the selected experimental domain (Figure 10). A similar interaction contour shape of process parameters’ interaction was observed in a previous study conducted in a microreactor using heterogeneous catalyst [2].

Based on the adopted optimization criteria, the highest desirability was obtained for the catalyst concentration of 0.1 g g^−1^, the residence time of 10 min, and methanol/oil volume ratio of 3:1. For those optimized process conditions, the predicted FAME content was 55.6%. The experiment with similar reaction conditions (slightly lower methanol/oil volume ratio of 2.25:1, the residence time of 10 min, and catalyst concentration of 0.1 g g^−1^) resulted in the FAME content of 51.2%.

For comparison, an experiment was performed in a stirred batch reactor, applying adequately comparable conditions. Using the same catalyst and oil and the following reaction conditions: temperature of 60 °C, catalyst concentration of 8% (in relation to oil), methanol/oil molar ratio of 12:1, and stirring speed of 800 rpm, a FAME content of only 18.6% was obtained in 10 min. This result confirmed that the microreactor system was very successful in intensifying the heterogeneously catalyzed transesterification process.

## 4. Conclusions

Statistical optimization and DoE were conducted for the transesterification reaction catalyzed by the waste chicken eggshell-based catalyst under mild reaction conditions in a custom-made microreactor. The catalyst was obtained from the waste sources and synthesized in a simple manner through the hydration–dehydration procedure and subsequent calcination at 600 °C. The catalyst thus obtained had a moderately developed specific surface area, good porosity, and adequate average pore diameter (17 nm), with evenly distributed CaO active phase. The response surface methodology combined with the Box–Behnken design was adopted due to its time-saving experimental matrix and proven adequacy for similar process systems. The FAME content was chosen as a process dependent variable, while residence time, catalyst concentration, and methanol/oil volume ratio were selected as independent process variables based on their major effects on the system. The statistical modeling of the microreactor transesterification resulted in a reduced quadratic model that linked FAME content with statistically significant terms. ANOVA analysis showed that the process parameters influenced the FAME content in descending order—catalyst concentration, residence time, and methanol/oil volume ratio—with the influence of the first two being similar and significant, while the influence of methanol/oil volume ratio was small. The optimal continuous reaction conditions in microreactor in the selected experimental domain were: catalyst concentration of 0.1 g g^−1^, residence time of 10 min, and methanol/oil volume ratio of 3:1. The microchannel clogging by the heterogeneous catalyst particles was not noticed. The maximum FAME content of 51.2% was obtained in the experiment and the reason for the lower yield was the deposition of the fine catalyst particles in the feed stream, which consisted of the catalyst suspension in oil. An attempt to solve the problem with the ultrasonic treatment of the suspension before the reaction did not give a significant result, because the precipitation began immediately after the cessation of treatment. The solution could be sought in the ultrasonic treatment of the entire microreactor system. Finally, obtaining biodiesel in a microreactor using CaO-based catalyst appears to be a promising process due to the very fast reaction rate under mild reaction conditions (several times faster than in a comparative commonly used batch reactor system), use of a heterogeneous waste-based catalyst, and the ability to easily scale-up the process.

## Figures and Tables

**Table 1:** Texture properties obtained by Hg-porosimetry and N_2_-physisorption.

	ES-R	ES-900	ES-HC-600
*S*_BET_ (m^2^ g^−1^)	<1	<1	19.3
*D*_p,av,BJH_ (nm)	8.7	28.0	17.0
*V*_p,BJH_ (mm^3^ g^−1^)	0.9	0.7	121.2
*P* (vol. %)	29.8	55.2	73.6

**Table 2:** The experimental matrix of the applied factorial design and the comparison of actual and predicted values of fatty acid methyl esters (FAME) content using the reduced quadratic model.

Run No.	Residance Time (*x*_1_), (min)	Catalyst Concentration (*x*_2_), (g g^−1^)	Methanol/oil Volume Ratio (*x*_3_)	FAME Content (*y*), (%)		Relative Deviation (%)
				Actual	Predicted	
1	7	0.1	3	28.48	33.6	−18.0
2	4	0.075	3	9.14	7.6	17.2
3	7	0.05	3	8.91	8.4	5.5
4	7	0.1	1.5	30.57	28.0	8.5
5	10	0.05	2.25	9.04	9.4	−4.3
6	10	0.1	2.25	51.12	49.0	4.2
7	7	0.05	1.5	4.81	2.8	42.3
8	10	0.075	3	32.02	29.0	9.6
9	7	0.075	2.25	6.43	7.5	−16.2
10	7	0.075	2.25	7.63	7.5	2.1
11	4	0.1	2.25	13.62	13.2	2.9
12	4	0.05	2.25	0.28	2.4	−760.7
13	4	0.075	1.5	2.08	1.9	7.8
14	10	0.075	1.5	18.51	23.3	−25.9
15	7	0.075	2.25	8.36	7.5	10.6

Mean relative percentage deviation (MRPD) value was ±11.3%, excluding the result of run 12.

**Table 3:** Analysis of variance (ANOVA) assesment for the reduced quadratic model.

Source	Sum of Squares	Degrees of Freedom	Mean Square	*F*-Value	*p*-Value
Model	2712.83	7	387.55	32.41	<0.0001
*x*_1_ (Residence time)	915.28	1	915.28	76.54	<0.0001
*x*_2_ (Catalyst concentration)	1268.82	1	1268.82	106.11	<0.0001
*x*_3_ (Methanol/oil volume ratio)	63.73	1	63.73	5.33	0.0543
*x*_1_ *x*_2_	206.5	1	206.5	17.27	0.0043
*x*_1_^2^	63.39	1	63.39	5.3	0.0548
*x*_2_^2^	175.71	1	175.71	14.69	0.0064
*x*_3_^2^	53.9	1	53.9	4.51	0.0714
Residual	83.7	7	11.96		
Lack of fit	81.8	5	16.36	17.23	0.0558
Pure error	1.9	2	0.9496		
Total correction	2796.54	14			

*R*^2^ = 0.97, *R*_adj_^2^ = 0.94, *R*_pred_^2^ = 0.84, *C.V.* = 22.45%, and Adequate precision = 18.64.

## Data Availability

The data presented in this study are available in insert article here.
